# Vasopressin versus norepinephrine in septic shock: a propensity score matched efficiency retrospective cohort study in the VASST coordinating center hospital

**DOI:** 10.1186/s40560-018-0344-2

**Published:** 2018-11-16

**Authors:** James A. Russell, Hugh Wellman, Keith R. Walley

**Affiliations:** 1 Centre for Heart Lung Innovation, St. Paul’s Hospital, University of British Columbia, 1081 Burrard Street, Vancouver, BC V6Z 1Y6 Canada; 2 Division of Critical Care Medicine, St. Paul’s Hospital, University of British Columbia, 1081 Burrard Street, Vancouver, BC V6Z 1Y6 Canada; 3 GenomeDx Biosciences Inc., 1038 Homer Street, Vancouver, BC V6B 2W9 Canada

**Keywords:** Septic shock, Vasopressin, Norepinephrine, Propensity match, Efficiency, Mortality, VASST

## Abstract

**Purpose:**

It is not clear whether vasopressin versus norepinephrine changed mortality in clinical practice in the Vasopressin and Septic Shock Trial (VASST) coordinating center hospital after VASST was published. We tested the hypothesis that vasopressin changed mortality compared to norepinephrine using propensity matching of vasopressin to norepinephrine-treated patients in the VASST coordinating center hospital before (SPH1) and after (SPH2) VASST was published.

**Methods:**

Vasopressin-treated patients were propensity score matched to norepinephrine-treated patients based on age, APACHE II, respiratory, renal, and hematologic dysfunction, mechanical ventilation status, medical/surgical status, infection site, and norepinephrine dose. The propensity score estimated the probability that a patient would have received vasopressin given baseline characteristics. For sensitivity analysis, we then excluded patients who had underlying severe congestive heart failure. The primary outcome was 28-day mortality.

**Results:**

Vasopressin- and norepinephrine-treated patients were similar after matching in SPH1 (pre-VASST); vasopressin-treated patients (*n* = 158) had a significantly higher mortality than norepinephrine-treated patients (*n* = 158) (60.8 vs. 46.2%, *p* = 0.009). In SPH2 after matching, the 28-day mortality rates were not significantly different; 31.2% and 26.9% in the vasopressin (*n* = 93) and norepinephrine (*n* = 93) groups, respectively (*p* = 0.518). The day 1 vasopressin dose in SPH1 vs. SPH2 was 0.036 units/min (SD 0.009) vs. 0.032 units/min (SD 0.005), *p* = 0.001, significantly lower in SPH2 after VASST.

**Conclusions:**

Before VASST, vasopressin use was associated with increased mortality compared to norepinephrine in the VASST coordinating center hospital. After VASST, there was no difference in mortality between vasopressin- and norepinephrine-treated patients. This may be the first retrospective propensity-matched cohort study of a sepsis treatment in a center that had previously coordinated a large pivotal randomized controlled trial of that treatment and could be a useful approach for other sepsis therapies.

**Trial registration:**

Registration: ISRCTN94845869

**Electronic supplementary material:**

The online version of this article (10.1186/s40560-018-0344-2) contains supplementary material, which is available to authorized users.

## Introduction

Vasopressin is deficient in septic shock [1, 2] and low-dose vasopressin infusion decreased norepinephrine dose requirements and organ dysfunction in early uncontrolled [3, 4] and controlled studies that were not powered for mortality [5]. The VASST trial (Vasopressin and Septic Shock Trial) [6] was a randomized blinded controlled trial of vasopressin vs. norepinephrine in septic shock powered for mortality. Although there was no overall statistically significant difference in mortality, some authors [7] and the Surviving Sepsis Campaign (SSC) [8] recommend the use of vasopressin in patients who are not responsive to norepinephrine. The Vasopressin vs. Norepinephrine as Initial Therapy in Septic Shock (VANISH) randomized controlled trial of vasopressin vs. norepinephrine used a higher dose and applied vasopressin earlier in septic shock but found no difference in acute kidney injury (the primary endpoint) or mortality [9] but did observe a reduction in the use of renal replacement therapy in vasopressin-treated patients. Efficacy trials such as VASST and VANISH should be followed by effectiveness studies to further assess whether publication of high-quality data alters practice and clinical outcomes.

Physicians have often been slow to adapt practice as new evidence is reported resulting in decreased compliance with guidelines and recommendations. It is possible that physicians would adapt practice more quickly and more widely in the centers that coordinated large pivotal trials that were incorporated into guidelines.

Studies of vasopressin use in clinical practice are limited. Physicians’ indications for use of vasopressin vary widely, and there is potential overuse of vasopressin in a survey of American intensivists [10]. Vail et al. [11] found wide variability (5–20% use) in clinical use of vasopressin in septic shock across the USA, suggesting local institution policies and physician beliefs drive use of vasopressin [12]. Neither of these studies evaluated mortality. One observational study (not matched) found no difference in vasopressin- vs. norepinephrine-treated patients’ mortality but did not address the period prior to or after VASST [13].

To date, there have been no efficiency studies comparing the mortality rates of vasopressin- vs. norepinephrine-treated septic shock patients in practice before and after the VASST results were known in the VASST coordinating center hospital. Thus, it is not clear whether vasopressin use changed and whether vasopressin alters mortality compared to norepinephrine in clinical practice. We took advantage of our being the VASST coordinating center hospital to test the hypothesis that vasopressin alters mortality compared to norepinephrine in septic shock using a propensity-matched cohort of patients treated clinically in a tertiary care center in the periods before and after the publication of VASST [6] in the VASST coordinating center hospital.

## Methods

### General

This study adheres to the STROBE Guidelines.

### Ethics

The retrospective observational and de-identified SPH cohort studies (SPH1 and SPH2) were approved by the St. Paul’s Hospital ethics committee who agreed that no patient consent was necessary.

### Patient cohorts

#### Inclusion criteria

This was a single university-affiliated tertiary care center study of use of vasopressin in the center that was the coordinating center of VASST. Patients admitted to the intensive care unit (ICU) of St. Paul’s Hospital in Vancouver, BC, Canada, who had two of four SIRS criteria who had suspected or proven infection and who were unresponsive to fluid resuscitation and received infusion of norepinephrine or vasopressin were included. Vasopressin and norepinephrine were given according to local clinical practice, i.e., choice of therapy was not randomized, controlled, or blinded. Less severe septic shock was defined as treatment with < 15 μg/min norepinephrine, and more severe septic shock was defined as treatment with ≥ 15 μg/min norepinephrine (the same definition as was used in VASST [6]).

Patients recruited prior to publication of VASST (2001–2007) were included in the SPH1 cohort, and patients recruited following the publication of VASST (2008–2012) were included in the SPH2 cohort.

Vasopressin may have different effects in patients who have underlying severe congestive heart failure (CHF) New York Heart Association IV (NYHA IV), so for sensitivity analysis, we excluded patients who had NYHA IV CHF in a separate analysis. We used the written section of the chart to determine whether patients had NYHA class IV heart failure. The diagnosis of heart failure was not confirmed by prior Echo, MUGA scan, or cardiac catheterization results. The self-reporting of symptoms at rest was based on histories obtained from the patients and their families.

### Matching vasopressin-treated to norepinephrine-treated patients

Vasopressin was likely given according to individual patient preference and likely varied widely [14]. Therefore, a well-matched control group is fundamental to the validity of this non-randomized study. Accordingly, the current study incorporated a robust, well-accepted matching strategy. After meeting the eligibility criteria, norepinephrine-treated patients were matched with vasopressin-treated patients using a computerized optimal matching algorithm incorporating baseline demographic and disease characteristics that had been identified a priori as likely influencing, first, the decision to prescribe vasopressin or, second, the probability of death. The number of matched control patients for each vasopressin-treated patient varied from one to three to increase the precision in the estimation of the differences between groups [15, 16].

Matching variables were baseline age, gender, APACHE II score, organ dysfunction (respiratory, renal, and coagulation), use of mechanical ventilation, underlying medical vs. surgical diagnosis, norepinephrine dose, and propensity score.

The matching strategy combined minimum-distance matching using “calipers” that force the matches for selected variables to fall within specified tolerances. A propensity score of the estimated probability that a patient would have received vasopressin given their key baseline characteristics was calculated, and patients were selected as matches had to be within a prespecified tolerance on this score. Combining the use of propensity scores with covariate matching is superior to the use of either strategy alone [17]. Individual variables were used to compute a multivariate distance (Mahalanobis distance). The Mahalanobis distance is a statistical measure of the distance between a point P and a distribution D and so measures how many standard deviations a point P is from the mean of the distribution D [18]. The Mahalanobis distance measures the number of standard deviations from P to the mean of D.

A propensity score of the estimated probability that a patient would have received vasopressin given their key baseline characteristics was calculated because combining both the propensity score and covariate matching is superior to the use of either strategy alone [19]. The intended clinical variables for the calculation of the Mahalanobis distance and the reasons these variables are shown in Additional file 1: Table S1.

The propensity score was estimated using a logistic regression model for the treatment group using the matching variables included in the calculation of Mahalanobis distances. Calipers were applied to selected key variables to ensure close matches. For age, a maximum 5-year difference was chosen. The propensity score caliper was set at 0.6 standard deviations (of the average propensity score) because this often decreases bias [20–22].

### Outcomes

The primary outcome variable was 28-day mortality.

### Statistical analyses

Baseline characteristics of vasopressin- and norepinephrine-treated patients were compared using parametric procedures (independent *t* test), non-parametric procedures (Wilcoxon rank sum test), or the Fisher exact test as appropriate. The primary analysis comparing 28-day mortality between the two treatment groups was performed using an unadjusted chi-square test according to treatment received. Conditional logistic regression was done to adjust for any baseline characteristics that remained significantly different (*p* < 0.05) between vasopressin- and norepinephrine-treated patients. Results are presented as absolute and relative risks and 95% confidence intervals. Kaplan-Meier curves describing the estimated probability of survival in the two treatment arms as a function of time from admission into the study were compared using the log-rank test statistic. Statistical significance was noted for *p* < 0.05.

## Results

### Overall SPH1 (pre-VASST)

In SPH1, there were 165 vasopressin-treated and 558 norepinephrine-treated patients before matching; at baseline, the vasopressin-treated patients were significantly younger, had higher APACHE II, and had more frequent renal, coagulation, and hepatic dysfunction, more often had underlying chronic disease and had a lower dose of norepinephrine (Table 1). After matching, vasopressin-treated (*n* = 158) and norepinephrine-treated (*n* = 158) patients were well matched (Table 1).

**Table 1:** Baseline characteristics and mortality before and after matching in SPH1

Variable	Norepinephrine	Vasopressin	*p*
Baseline characteristics before matching of norepinephrine- vs. vasopressin-treated patients in SPH1			
Age (years), *X* ± SD	60.7 ± 16.2	56.1 ± 15.7	< 0.001
APACHE II, *X* ± SD	25.6 ± 8.0	28.8 ± 8.9	< 0.001
Gender (% male)	61.8	73.3	0.007
Surgical (%)	39.2	35.2	0.146
Respiratory (%)	88.3	92.1	0.17
Renal (%)	68.1	81.2	< 0.001
Coagulation (%)	23.3	36.4	< 0.001
Hepatic (%)	14.3	22.4	0.013
Neurological (%)	64.7	79.4	< 0.001
Ventilated (%)	92.7	89.1	0.142
Any chronic disease (%)	44.8	55.8	0.013
Norepinephrine dose (ug/min)	13.2 ± 14.8	21.4 ± 21.7	< 0.001
Baseline characteristics after matching of norepinephrine- vs. vasopressin-treated patients in SPH1			
Age (years), *X* ± SD	57.1 ± 15.1	56.4 ± 15.4	0.65
APACHE II, *X* ± SD	28.1 ± 8.3	28.4 ± 8.4	0.782
Gender (% male)	67.1	72.8	0.27
Surgical (%)	34.8	34.8	1
Respiratory (%)	93.0	91.8	0.671
Renal (%)	74.7	81.0	0.176
Coagulation (%)	28.5	36.1	0.149
Hepatic (%)	18.4	22.2	0.401
Neurological (%)	69.0	78.5	0.055
Ventilated (%)	94.3	89.2	0.101
Any chronic disease (%)	51.9	54.4	0.652
Norepinephrine dose (ug/min)	15.9 ± 16.7	19.8 ± 18.9	0.139
Mortality of norepinephrine- vs. vasopressin-treated patients in SPH1			
28-day mortality (%)	46.2	60.8	0.009

There was a significantly higher mortality rate in the vasopressin- (60.8%) vs. norepinephrine-treated patients (46.2%), *p* = 0.009 (Table 1) in SPH1. This difference in mortality represents an absolute risk reduction of 14.6% and a number needed to treat to save one life of 6.8.

### Sensitivity analysis—exclusion of patients who had NYHA IV CHF in SPH1 (pre-VASST)

We excluded patients who had underlying NYHA IV CHF in SPH1. There were 145 vasopressin-treated patients and 525 norepinephrine-treated before matching (Table 2). Before matching, vasopressin-treated patients were significantly younger, had higher APACHE II scores, were more frequently male, had more frequent renal, coagulation, hepatic, and CNS dysfunction, and were receiving a higher dose of norepinephrine at baseline (Table 2). After matching, there were 140 vasopressin-treated and 140 norepinephrine-treated patients (Table 2). The matching strategy and technique was quite successful; after matching, vasopressin- and norepinephrine-treated patients were well matched (Table 2).

**Table 2:** Baseline characteristics and mortality of norepinephrine- vs. vasopressin-treated patients in SPH1 before and after matching. Sensitivity analysis after exclusion of patients who had underlying NYHA IV CHF

Variable	Norepinephrine	Vasopressin	*p*
Baseline characteristics norepinephrine- vs. vasopressin-treated patients in SPH1 before matching			
Age (years), *X* ± SD	60.2 ± 16.3	55.5 ± 15.5	< 0.001
APACHE II, *X* ± SD	25.5 ± 8.0	29.0 ± 8.7	< 0.001
Gender (% male)	61.3	73.1	0.009
Surgical (%)	28.2	31.0	0.503
Respiratory (%)	88.6	92.4	0.183
Renal (%)	68.4	82.8	< 0.001
Coagulation (%)	23.4	38.0	< 0.001
Hepatic (%)	13.9	22.8	0.01
Neurological (%)	64.8	80.0	< 0.001
Ventilated (%)	92.4	89.0	0.188
Any chronic disease (%)	41.3	49.7	0.073
Norepinephrine dose (ug/min)	13.6 ± 15.0	21.8 ± 22.0	< 0.001
Baseline characteristics norepinephrine- vs. vasopressin-treated patients in SPH1 after matching			
Age (years), *X* ± SD	56.8 ± 14.9	56.1 ± 15.3	0.644
APACHE II, *X* ± SD	28.5 ± 8.2	28.8 ± 8.4	0.831
Gender (% male)	66.4	72.9	0.242
Surgical (%)	31.4	31.4	1
Respiratory (%)	92.9	92.1	0.821
Renal (%)	77.1	82.1	0.299
Coagulation (%)	28.6	37.9	0.099
Hepatic (%)	18.6	23.6	0.305
Neurological (%)	71.4	79.3	0.127
Ventilated (%)	93.6	89.3	0.2
Any chronic disease (%)	47.9	48.6	0.905
Mortality of norepinephrine- vs. vasopressin-treated patients in SPH1 after matching			
28-day mortality (%)	46.4	62.9	0.006

After excluding patients who had NYHA IV CHF and then matching, the vasopressin-treated mortality remained significantly higher (62.9%) than that of the norepinephrine-treated patients (46.4%) (*p* = 0.006 unadjusted; *p* = 0.02 adjusted) (Table 2).

### Overall SPH 2 (post-VASST)

In SPH2, there were 525 vasopressin-treated and 145 norepinephrine-treated patients before matching; at baseline, the vasopressin-treated patients had higher APACHE II and norepinephrine dose (Table 3). After matching, there was no difference in any baseline characteristic between vasopressin-treated (*n* = 93) and norepinephrine-treated (*n* = 93) patients (Table 3).

**Table 3:** Baseline characteristics and mortality of patients who had septic shock according to norepinephrine- vs. vasopressin-treated infusion prior to and after matching in SPH2

Variable	Norepinephrine	Vasopressin	*p*
Baseline characteristics norepinephrine- vs. vasopressin-treated patients prior to matching in SPH2			
Age (years), *X* ± SD	61.4 ± 16.8	60.9 ± 13.9	0.574
APACHE II, *X* ± SD	21.1 ± 7.2	23.9 ± 6.4	< 0.001
Gender (% male)	66.4	62.4	0.617
Surgical (%)	21	22.6	0.761
Renal (%)	18.2	22.6	0.376
Hepatic (%)	10.2	15.1	0.232
Neurological (%)	36.5	32.3	0.48
Ventilated (%)	82.7	82.8	0.985
Any chronic disease (%)	45.3	50.5	0.401
Norepinephrine dose (ug/min)	*14.2 ± 13.6*	*19.2 ± 5.9*	*< 0.001*
Baseline characteristics norepinephrine- vs. vasopressin-treated patients after matching in SPH2			
Age (years), *X* ± SD	61.4 ± 14.5	60.9 ± 13.9	0.812
APACHE II, *X* ± SD	23.8 ± 6.5	23.9 ± 6.4	0.939
Gender (% male)	30.1	37.6	0.278
Surgical (%)	19.3	22.6	0.589
Renal (%)	20.4	22.6	0.721
Hepatic (%)	15.1	15.1	1
Neurological (%)	40.3	32.3	0.13
Ventilated (%)	88.2	82.8	0.298
Any chronic disease (%)	50.5	50.5	1
Norepinephrine dose (ug/min)	16.9 ± 14.6	19.2 ± 15.9	0.242
Mortality of norepinephrine- vs. vasopressin-treated patients after matching in SPH2			
28-day mortality (%)	26.9	31.2	0.518

In SPH2, the 28-day mortality rates were much lower than in SPH1 and not significantly different between vasopressin-treated and norepinephrine-treated patients, 26.9% and 31.2% in the norepinephrine and vasopressin groups, respectively (*p* = 0.518) (Table 3).

We compared the vasopressin doses of SPH1 vs. SPH2 and found that day 1 vasopressin dose in SPH 1 vs. SPH2 was 0.036 units/min (SD 0.009) vs. 0.032 units/min (SD 0.005), *p* = 0.001, significantly lower in SPH2 (Additional file 1: Figure S1).

### Sensitivity analysis—exclusion of patients who had NYHA IV CHF in SPH2 (post-VASST)

We excluded patients who had underlying NYHA IV CHF in SPH2. There were 93 vasopressin-treated patients and 214 norepinephrine-treated before matching (Table 4). Before matching, vasopressin-treated patients had significantly higher APACHE II scores and were receiving a higher dose of norepinephrine at baseline (Table 4). After matching, there were 93 vasopressin-treated and 93 norepinephrine-treated patients (Table 4). After matching, there were no significant differences in baseline characteristics between the vasopressin- vs. norepinephrine-treated patients (Table 4). After excluding patients who had NYHA IV CHF and then propensity matching, the mortality rates were lower in SPH1 and there was no significant difference in 28-day mortality rates of the vasopressin-treated (31.2%) vs. the norepinephrine-treated (26.9%) patients (*p* = 0.52 unadjusted; *p* = 0.49 adjusted) (Table 4).

**Table 4:** Baseline characteristics and mortality of norepinephrine- vs. vasopressin-treated patients before and after matching in SPH2. Sensitivity analysis after exclusion of patients who had underlying NYHA IV CHF

Variable	Norepinephrine	Vasopressin	*p*
Baseline characteristics norepinephrine- vs. vasopressin-treated patients before matching in SPH2			
Age (years), *X* ± SD	61.4 ± 16.8	60.9 ± 13.9	0.574
APACHE II, *X* ± SD	21.1 ± 7.2	23.9 ± 6.4	< 0.001
Gender (% male)	66.4	62.4	0.617
Surgical (%)	21.0	22.6	0.761
Renal (%)	18.2	22.6	0.376
Hepatic (%)	10.3	15.1	0.232
Neurological (%)	36.5	32.3	0.48
Ventilated (%)	82.7	82.8	0.985
Any chronic disease (%)	45.3	50.5	0.401
Norepinephrine dose (ug/min)	14.2 ± 13.6	19.2 ± 15.9	< 0.001
Baseline characteristics norepinephrine- vs. vasopressin-treated patients after matching in SPH2			
Age (years), *X* ± SD	61.4 ± 14.5	60.9 ± 13.9	0.812
APACHE II, *X* ± SD	23.8 ± 6.5	23.9 ± 6.4	0.939
Gender (% male)	69.9	62.4	0.278
Surgical (%)	19.3	22.6	0.589
Renal (%)	20.4	22.6	0.721
Hepatic (%)	15.1	15.1	1
Neurological (%)	43	32.3	0.13
Ventilated (%)	88.2	82.8	
Any chronic disease (%)	50.5	50.5	1
Norepinephrine dose (ug/min)	16.9 ± 14.6	19.2 ± 15.9	0.242
Mortality of norepinephrine- vs. vasopressin-treated patients after matching in SPH2			
28-day mortality (%)	26.9	31.2	0.518

## Discussion

In this single center—VASST coordinating center hospital—propensity-matched retrospective cohort study of patients who had septic shock, patients treated with vasopressin had significantly higher mortality than norepinephrine-treated patients in the period before VASST [6] was published. After the publication of VASST, there was no difference in mortality between vasopressin- and norepinephrine-treated patients. This suggests—but does not prove—there may have been a change in vasopressin prescribing and a change in vasopressin- vs. norepinephrine treatment-related outcomes. To our knowledge, this is the first propensity-matched retrospective cohort study of a sepsis treatment in a center that had previously coordinated a large pivotal randomized controlled trial of that treatment.

We used propensity matching on several key variables to mitigate bias based on selection of patients who received vasopressin. The logical basis for matching was first to simulate in a non-randomized population a vasopressin-treated and non-vasopressin-treated (control) group that is as comparable as possible at baseline and so that differences in outcomes can be better attributed to the vasopressin treatment or not. The logic of the specific matching variables was to match for variables that are associated with use of vasopressin: age, gender, APACHEII score, organ dysfunction (respiratory, renal, and coagulation), use of mechanical ventilation, underlying medical vs. surgical diagnosis, and norepinephrine dose.

Heart failure patients were excluded because severe heart failure (NYHA class IV) is a contraindication to use of vasopressin because vasopressin can decrease cardiac output. In a clinical observational cohort study, some patients with heart failure could have received vasopressin and could have been worsened by vasopressin-induced decrease in cardiac output. Thus, the exclusion of heart failure patients addresses potential bias (overestimation of mortality in vasopressin-treated patients) and adds robustness to the study. The use of inotropic agents was not the reason to exclude heart failure patients.

After excluding patients who had NYHA IV CHF and then propensity matching, there was a difference in the 28-day mortality between the norepinephrine-treated patients of SPH1 (46.4%) and those of SPH2 (26.9%) (Tables 2 and 4) that may be due to the difference in severity of illness. In SPH1, APACHE II score was 28.5 ± 8.2 in the norepinephrine group and 28.8 ± 8.4 in the vasopressin group, whereas in SPH2, APACHE II score was 23.8 ± 6.5 in the norepinephrine group and 23.9 ± 6.4 in the vasopressin group (Tables 2 and 4).

We do not know the cause of mortality difference between SPH1 and SPH2, and there may be factors related to mortality other than the dose of vasopressin. We speculate that changes in availability of ICU beds or referrals from the emergency, and other sites may have resulted in a change in severity of illness and mortality of SPH 2 compared with SPH1.

We clarify that the sepsis 2.0 definition was used in the original VASST trial inclusion and exclusion criteria [23]. Thus, we also used this definition in our current retrospective cohort study so that we could compare the results of VASST to the results of our retrospective cohort study.

Since Landry and colleagues’ [1, 2] discovery of a vasopressin deficiency in septic shock, subsequent small uncontrolled [3, 4] or controlled trials [5] were the available evidence, and the use of vasopressin was uncertain in septic shock. The VASST randomized controlled trial showed that there was no overall difference in mortality between vasopressin- and norepinephrine-treated patients [6]. However, in the stratum of patients who had less severe shock (norepinephrine infusion less than 15 μg/min at time of randomization), there was a very strong trend to decreased mortality in the vasopressin compared to the norepinephrine-treated group (*p* = 0.05). So, skeptics interpreted that there was no benefit of vasopressin, some authors [7, 24, 25] and the Surviving Sepsis Campaign [8, 26] recommended vasopressin for patients not responding to norepinephrine, and others likely used vasopressin in patients who had less severe shock (based on the VASST stratum results). The authors have been on record as recommending 0.01–0.04 units/min vasopressin in patients with more severe shock and that we recommend starting vasopressin earlier when patients have less severe shock because this is the subgroup that appeared to have benefit in the original VASST analysis and in the retrospective analyses that used the sepsis 3.0 definition [15, 23]. One meta-analysis suggested efficacy of vasopressin vs. norepinephrine in septic shock [16].

The VANISH randomized controlled trial used a higher dose of vasopressin and applied vasopressin earlier than did VASST, but also found no difference in acute kidney injury (the primary endpoint of VANISH) or mortality of vasopressin- vs. norepinephrine-treated patients [9].

Randomized controlled trials (such as VASST and VANISH) assess efficacy of a drug in a highly selected group of patients carefully selected to test whether the drug can decrease the primary endpoint under trial conditions. Efficacy trials should be followed by effectiveness trials to better assess benefits and risks of drugs such as vasopressin in the broader range of patients in clinical practice. Indeed, despite inherent methodological limitations (lack of randomization and blinding), comparative effectiveness research is ramping up in the USA [19, 27, 28] and has shown effectiveness of drugs and devices (e.g., drug-eluting stents [29]) used in practice.

Physicians may not widely adapt new evidence into clinical practice when new evidence is reported so outcomes do not improve as soon or as well as expected. We speculated that physicians would alter vasopressin use quickly and widely in the VASST coordinating center hospital after the VASST results were known. Furthermore, there had been no propensity-matched cohort studies of use of vasopressin vs. norepinephrine in septic shock before and after the publication of VASST [6]. In our current study, before VASST was published, vasopressin was associated with increased mortality compared to norepinephrine and our study suggests—but does not prove—that after publication of VASST, physicians were more selective in prescribing vasopressin such that the difference in mortality between vasopressin- and norepinephrine-treated patients disappeared after VASST. This interpretation is supported by the significantly lower vasopressin dose used after VASST (SPH2) than before VASST (SPH1). Finally, if we assume that randomized controlled trial results align most closely with the true effects of vasopressin in septic shock, then it is satisfying to see that our propensity-matched efficiency study post-VASST aligned well with the overall negative results of the two large randomized controlled trials of vasopressin in septic shock, VASST [23] and VANISH [9]. These randomized controlled trials found no difference in mortality between vasopressin and norepinephrine.

But one could ask how the current study could change physicians’ practice. The current study is important because it validates the results of VASST in clinical practice, albeit in the VASST coordinating center hospital. It is an important first step in moving from the VASST efficacy trial to an efficiency trial in the VASST coordinating center, which could be followed by a broader multi-center efficiency trial to compare vasopressin versus norepinephrine in clinical practice of septic shock. We speculate that our current study results could apply to other hospitals that also use vasopressin vs. norepinephrine in septic shock.

How did the clinical equipoise regarding vasopressin in septic shock translate into practice in other studies? There is indirect evidence of possible overuse of vasopressin in practice based on a survey of US intensivists’ vasopressor preferences [14]. There is also very wide inter-institutional variation in the use of vasopressin for septic shock in the USA; mean hospital use of vasopressin was 12%, with a range of 0 to 70% [11]. Lower age and respiratory dysfunction were clinical features associated with use of vasopressin as was hospital of admission [11].

Although the reasons for changes in mortality rates of vasopressin vs. norepinephrine are not known, one possible explanation was the use of higher doses of vasopressin in the VASST coordinating center hospital before VASST than after VASST was known. The day 1 vasopressin dose in SPH 1 vs. SPH2 was 0.036 units/min (SD 0.009) vs. 0.032 units/min (SD 0.005), *p* = 0.001, significantly lower in SPH2. In VASST, the blinded vasopressin infusion was started at 0.01 units/min and titrated to a maximum of 0.03 units/min [23] while in VANISH, the vasopressin dose was up to 0.06 units/min [9]. Higher dose vasopressin is associated with increased risk of adverse events [25, 30, 31] such as vital organ and digital ischemia, which may have contributed to increased mortality in our propensity-matched efficiency study. We were concerned about the difference in mortality between SPH1 and SPH2 because our study showed significantly lower vasopressin dose used after VASST than before VASST may have resulted in lower 28-day mortality. However, this interpretation is limited because of the severity and mortality differences between SPH1 and SPH2.

The differences in mortality between vasopressin-treated vs. non-vasopressin-treated patients in SPH1 vs. SPH2 could be related to the differences in vasopressin benefits (lowering norepinephrine dose, decreasing organ dysfunction) vs. side effects (cardiac, gut, digital and renal ischemia [23, 30], and arrhythmias) related to mechanisms such as effects of vasopressin on vascular tone, immune effects (such as cytokines) [32, 33], vascular permeability, renal blood flow and function [9, 34], and von Willebrand factor release. We did not assess these possible mechanisms in this clinical study.

The strengths of our study are efficiency evaluation of vasopressin vs. norepinephrine in the hospital that coordinated VASST, the quality of the matching that removed differences in baseline characteristics between vasopressin- and norepinephrine-treated patients, and sensitivity analysis by excluding patients who had New York Heart Association class IV congestive heart failure in a separate analysis.

The limitations of the study are the lack of control by randomization and blinding (so there could be remaining confounding by indication), the single-center design (the single-center design of our study limits generalizability), the use of the sepsis 2.0 (vs. the sepsis 3.0) definition in VASST, and the lack of control of secular changes in management of septic shock before and after VASST (but these would have had to favor vasopressin outcomes in some unknown way). We do not know how many patients were self-reported NYHA class IV heart failure but did not have confirmation such as by echocardiography or cardiac catheterization. The sample size was a convenience sample size of the available patients in the SPH and SPH2 cohorts. We only included patients who had all of the data required for the current study, and so there was no missing data. The difference in day 1 vasopressin dose between SPH1 vs. SPH2 (0.036 vs. 0.032 units/min) was statistically different (*p* = 0.001), but it is not entirely certain how much of a clinical impact this difference would have made on mortality. Another limitation is that we do not have other clinical variables including use of corticosteroids, need for positive pressure ventilation, time to first appropriate antibiotic, other concomitant vasopressor use, fluid volume during resuscitation, and transfusion(s).

## Conclusions

Before VASST was published, vasopressin may have increased mortality compared to norepinephrine in the VASST coordinating center hospital. After the VASST results were known, there was no difference in mortality between vasopressin- and norepinephrine-treated patients. This is the first propensity-matched cohort study of a sepsis treatment in a center that had previously coordinated a large pivotal randomized controlled trial of that treatment—this approach may be useful for other sepsis therapies.

## Additional file

Additional file 1: **Table S1.** Rationale for Mahalanobis distance variable selection in the propensity matching of vasopressin to norepinephrine-treated patients with septic shock. Figure S1. Doses of vasopressin in SPH1 and SPH2. The dose of vasopressin (mean ± SD) in SPH2 was significantly lower than that in SPH1 (*p* = 0.001). (DOCX 26 kb)

## Abbreviations

APACHE II: Acute Physiology and Chronic Health Evaluation II
CHF: Congestive heart failure
ICU: Intensive care unit
NYHA IV: New York Heart Association IV
SD: Standard deviation
SPH: St. Paul’s Hospital
SSC: Surviving Sepsis Campaign
USA: United States of America
VANISH: Vasopressin vs. Norepinephrine as Initial Therapy in Septic Shock
VASST: Vasopressin and Septic Shock Trial
