# Supplementary material for: Vasopressin versus norepinephrine in septic shock: a propensity score matched efficiency retrospective cohort study in the VASST coordinating center hospital
Source: J Intensive Care. 2018 Nov 16;6:73. doi: 10.1186/s40560-018-0344-2 (PMC6240281; doi:10.1186/s40560-018-0344-2)
Supplement: Supplementary file 1 — Table S1. Rationale for Mahalanobis distance variable selection in the propensity matching of vasopressin to norepinephrine-treated patients with septic shock. Figure S1. Doses of vasopressin in SPH1 and SPH2. The dose of vasopressin (mean ± SD) in SPH2 was significantly lower than that in SPH1 (p = 0.001). (DOCX 26 kb) [file 40560_2018_344_MOESM1_ESM.docx]

**Additional file 1**

**VASOPRESSIN OR NOREPINEPHRINE IN SEPTIC SHOCK?**

**A PROPENSITY SCORED MATCHED EFFICIENCY STUDY**

**JAMES A. RUSSELL MD (1,2), HUGH WELLMAN (3),**

**KEITH R. WALLEY, MD (1,2)**

**Table S1.** Rationale for Mahalanobis Distance Variable selection in the propensity matching of vasopressin to norepinephrine-treated patients with septic shock.

| **Baseline Characteristic**  **(Variable)** |  | **Associated with Mortality Risk Only** |  | **Associated with Vasopressin Selection and Mortality Risk** |  | **Comments** |
| --- | --- | --- | --- | --- | --- | --- |
|  |  |  |  |  |  |  |
| Age |  | √ |  | v |  | Increased age is associated with increased mortality . Age is a variable in APACHE II. Decreased age is associated with use of vasopressin. |
|  |  |  |  |  |  |  |
| APACHE II |  | **√** |  | **v** |  | APACHE II is associated with mortality and use of vasopressin |
|  |  |  |  |  |  |  |
| Cardiovascular organ dysfunction |  |  |  | **√** |  | Vasopressin may be effective in shock |
|  |  |  |  |  |  |  |
| Respiratory organ dysfunction |  | √ |  | **v** |  | Respiratory dysfunction increases mortality and increased organ dysfunction is associated with use of vasopressin. |
|  |  |  |  |  |  |  |
| Renal organ dysfunction |  | v |  | √ |  | Renal dysfunction increases mortality. Patients who had Risk (RIFLE category) of renal dysfunction had more beneficial response to vasopressin([43](#_ENREF_43)) |
|  |  |  |  |  |  |  |
| Hematologic organ dysfunction |  | **√** |  | **v** |  | Hematologic dysfunction increases mortality. Hematologic dysfunction could alter response to vasopressin (vasopressin increases von Willebrand multimers) |
|  |  |  |  |  |  |  |
| Use of mechanical ventilation |  | √ |  | **v** |  | Need for mechanical ventilation increases mortality and increased organ dysfunction is associated with use of vasopressin.. |
|  |  |  |  |  |  |  |
| Medical or surgical status |  | **√** |  | **v** |  | Medical vs. surgical admission is a variable in APACHE II. Vasopressin use is more common in medical patients. |
|  |  |  |  |  |  |  |
| Site of primary infection |  | **√** |  |  |  | Site of primary infection increase mortality([44](#_ENREF_44)). |
| Norepinephrine dose |  | **v** |  | **√** |  | High dose of norepinephrine is associated with higher mortality (VASST([27](#_ENREF_27))). Lower doses of norepinephrine are associated with increased response to vasopressin. |

**Figure S1.** Doses of vasopressin in SPH1 and SPH2. The dose of vasopressin (mean +/- SD) in SPH2 was significantly lower than in SPH1 (p = 0.001)
